# The continuing evolution of publishing in the biological sciences

**DOI:** 10.1242/bio.037325

**Published:** 2018-08-15

**Authors:** Steven Kelly

**Affiliations:** Department of Plant Sciences, University of Oxford, South Parks Road, Oxford OX1 3RB, UK


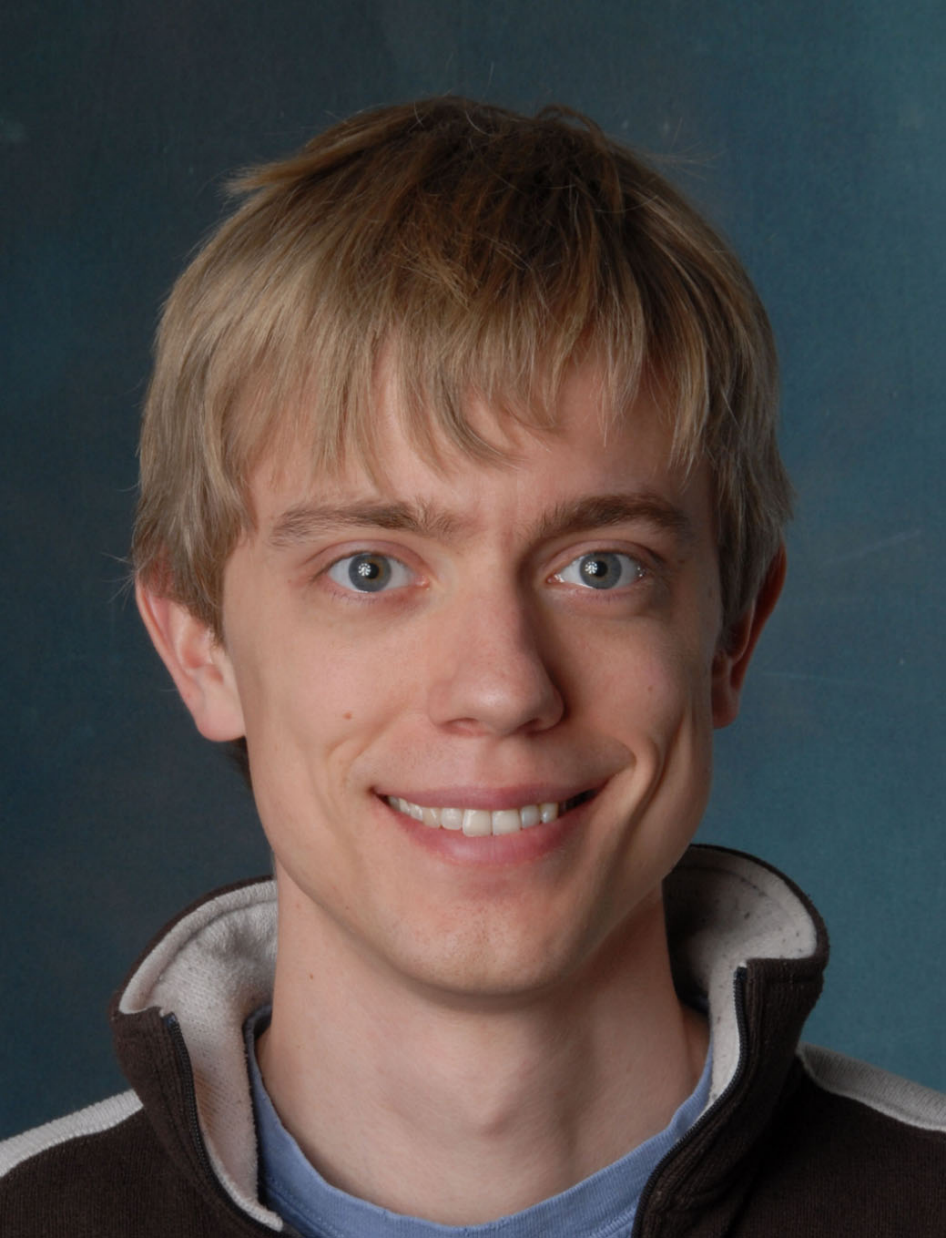


Following eight years at the helm of Biology Open (BiO), Founding Editor-in-Chief Jordan Raff this year made the decision to stand down from the role (Raff, 2018). As I take over from Jordan, I would like to reflect on the changing landscape of publishing in the biological/biomedical sciences since I started my scientific career as an undergraduate student in 1999. It will come as no surprise that, as a community, we are publishing more papers than ever before. Over the period between 2000 and 2017, the number of journal articles published annually increased by ∼3.6% per year (Fig. 1A), with over 400,000 papers published in 2017. What is perhaps unexpected is that the number of authors of those publications increased 50% faster over the same period (∼5.4% per year; Fig. 1B); in 2017, there were, on average, 6.2 authors per paper compared with 4.5 authors per paper in 2000 (Fig. 1C). This increase in the number of authors per paper is probably attributable to two main factors: first, it might be due to researchers finding more ways to work collaboratively, bringing together different expertise to address scientific questions. Second, it might be a sign that it is getting harder to publish, and thus the combined efforts of 38% more people are required to produce a publishable quantum of research in 2017 than in 2000.

**Fig. 1.**
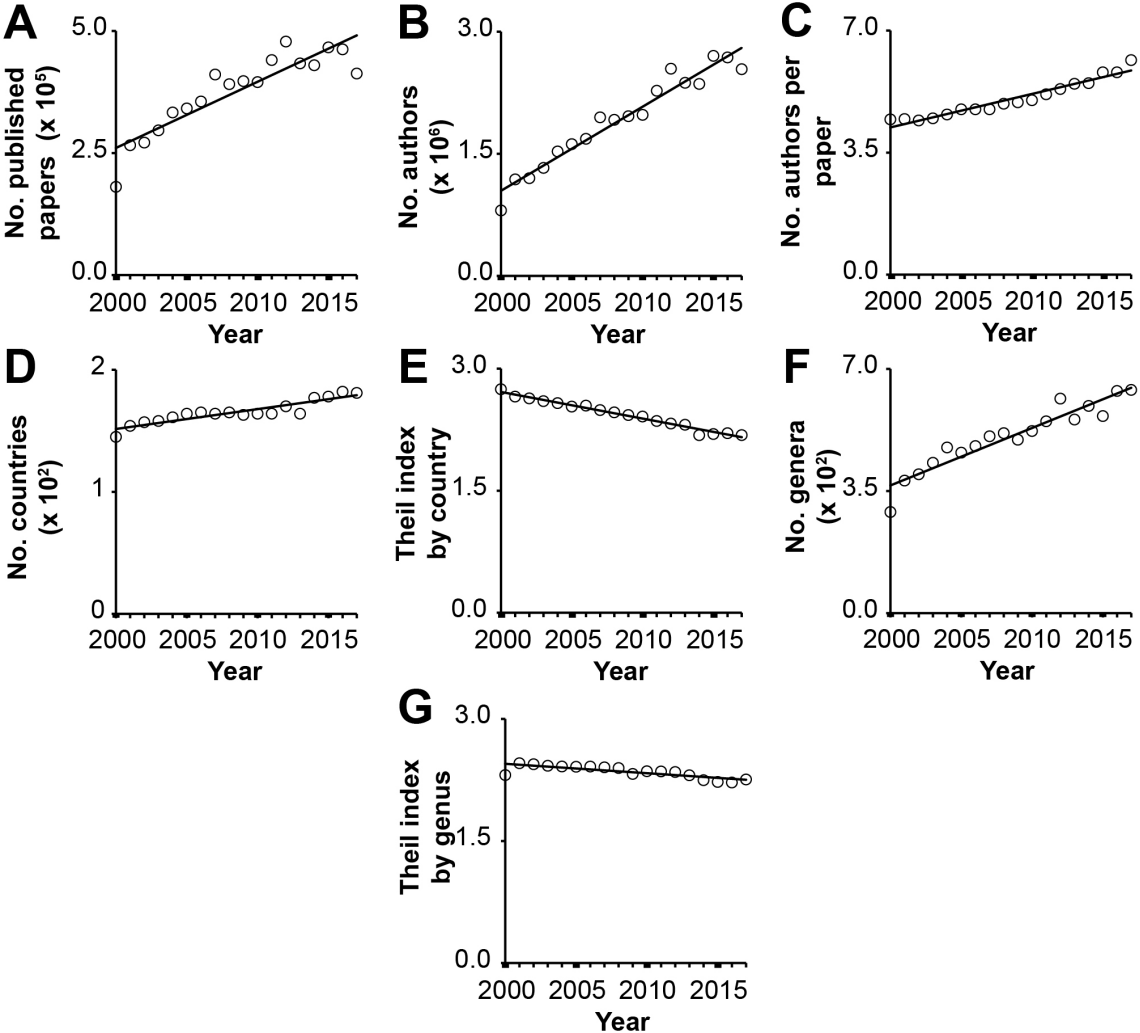
**Analysis of journal articles published between 1 January 2000 and 31 December 2017.** All publication data were downloaded from NCBI using the esearch/efetch commands from Entrez Direct E-utilities (Kans, 2010). (A) The number of papers published (R^2^=0.79, *P*<0.001). (B) The number of authors (R^2^=0.94, *P*<0.001). (C) The number of authors per paper (R^2^=0.93, *P*<0.001). (D) The number of countries listed in authors’ affiliations (R^2^=0.82, *P*<0.001). (E) The Theil index of international publication inequality (R^2^=0.98, *P*<0.001). (F) The number of genera listed in journal article abstracts (R^2^=0.89, *P*<0.001). (G) The Theil index of taxonomic inequality (R^2^=0.63, *P*<0.001). For simplicity's sake, all data were fit using simple linear models using the lm package in R (R Core Team, 2017). Adjusted R^2^ values with associated *P*-values are provided.

In addition to increasing our outputs, scientific publication in the biological/biomedical sciences is becoming more international. The number of countries listed in authors’ affiliations has increased by ∼1% per year (Fig. 1D) and the international inequality in scientific publication has decreased by ∼1.3% per year (Fig. 1E). Thus, the spread of scientific research is becoming more evenly distributed among countries around the world. As well as becoming more international, the focus of biological science research is also steadily expanding. One measure of this is the continued increase in the number of different genera that are listed in abstracts each year (∼3.2% per year; Fig. 1F). However, although the breadth continues to expand, the change in taxonomic inequality is not keeping pace (∼0.5% decrease per year, Fig. 1G). Thus, despite an increasing number of forays into alternative non-model species, traditional model organisms continue to be the focus of the majority of our research.

It is also clear from looking at the data that the kind of science practiced has evolved during this time. To illustrate this evolution, a phylogenetic tree constructed from the similarity of word content of abstracts shows a remarkably regular ‘ladder-like’ topology, with each successive year being most similar to its preceding year (Fig. 2A). To identify the key factors that are underpinning this change, the data were subject to cluster extraction to identify the cohorts of words that have gained and lost favour over this time. This identified two major clusters each of ∼7000 words that have steadily become either more or less prevalent in our abstracts over this time (Fig. 2B, Supplemental File 1). Inspection of these words revealed changes in the occurrence of experimental techniques, such as a decrease in the use of chromatography (Fig. 2C) and an increase in the use of spectrometry (Fig. 2D). It also revealed an increase in an abundance of words associated with analytical and quantitative approaches, as well as terms associated with interdisciplinary research (Fig. 2D). Thus, changes in the use of a broad range of techniques and approaches are fuelling the evolution of our science. What is perhaps most striking about this analysis of the evolution of biological/biomedical sciences is that the magnitude of the similarity between successive years has been increasing by ∼0.2% per year (R^2^=0.80, *P*<0.001). This is readily visible from the increase in ‘redness’ of the heatmap between successive years as time progresses (Fig. 2A). Thus, it appears that, despite the increases in the number of papers, the number of authors and the taxonomic breadth, the rate at which our science is evolving is slowing down. This is an inevitable manifestation of the law of diminishing returns, as not all discoveries can be expected to break new ground. However, they all need to be published and made freely accessible to all to advance our understanding of the natural world.

**Fig. 2.**
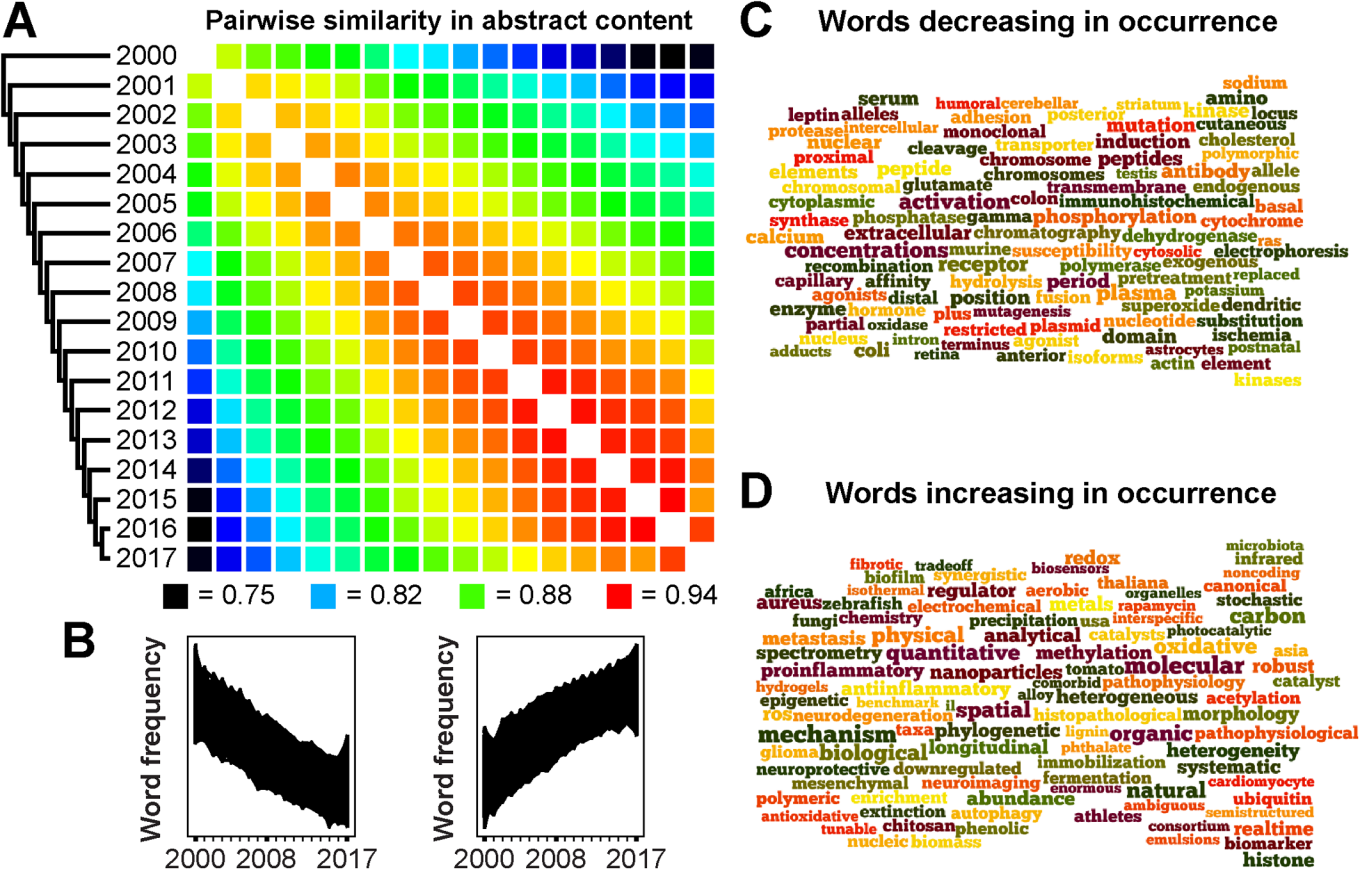
**Analysis of word content of journal article abstracts published between 1 January 2000 and 31 December 2017.** (A) Minimum evolution phylogenetic tree constructed from pairwise Spearman correlation coefficients (*ρ*^2^) of abstract word frequencies from each year. Correlation coefficients were converted to dissimilarity scores (distances) for phylogenetic inference using FastMe (Lefort et al. 2015). Distances between two years *α* and *β* were evaluated as *d*(*α*,*β*)=1–­*ρ*^2^. (B) The two largest clusters obtained from *clust* (Abu-Jamous and Kelly, 2018preprint) analysis of the abstract word frequency data. (C) Wordle (http://www.wordle.net/) of the 100 most frequently used words from the decreasing cluster. (D) Wordle of the 100 most frequently used words from the increasing cluster.

The clear trends in the data mean that it is relatively trivial to predict what scientific publishing will look like in 2040. Science will be more international; there will be more papers from more authors in more countries. We will be working even more collaboratively than we are now and the average paper will have approximately eight contributing authors. Although we will be publishing on an even larger range of taxa, we will still be focusing on the same tried-and-tested model organisms. Moreover, despite all these increases in breadth, collaboration and quantity, the science of 2040 will be more similar to the science we are doing today than contemporary science is to that of 2000.

Given that we can guess what scientific publication will look like, can we also speculate what scientific publishers will look like? Between 2000 and 2017, we saw the steady rise of Open Access publishing and the birth of preprints in the biological/biomedical sciences. In 2040, Open Access online-only journal platforms, such as BiO, will be all that remain. All subscription journals will move to this model or perish. It will also become the default that we submit preprints of our work while it is undergoing review, if not before. Both of these changes are essential to accelerate the pace of scientific discovery, and to continue to reduce scientific inequality around the globe. However, both need to be made while striving to continually improve the integrity, veracity and impartiality of published science.

It cannot be escaped that there are costs associated with publishing and distributing research. Even with relatively streamlined online journals, there are academic editors, reviewers, administrative personnel, servers and data management systems, all of which are required to process and support the peer-review and publication processes. Moreover, there is a commitment to provide access to these results in perpetuity. To provide this service to the scientific community, there are really only three options. The first is that commercial publishing companies will raise publication costs to offset subscription losses and maintain profit margins. As before, the funds raised from this will continue go to shareholders, significantly reducing the amount of money available for scientific research. The second option is that non-for-profit organisations such as learned societies and indeed, The Company of Biologists, publisher of Biology Open, will expand (or consolidate) their portfolio of Open Access journals, keeping publication costs low and returning any profits to the scientific community in the form of research, travel or conference grants. The third option is that funders will become the publishers of the future and set up Open Access platforms to disseminate their funded results. Such platforms have already begun to emerge, such as Open Research from the Wellcome Trust, and Gates Open Research from the Bill and Melinda Gates foundation. The question that we as authors need to ask is: where do we want our grant money to go? We can choose to give our money either to a commercial publishing company (who will in turn give it to its shareholders) or we can give it to a society or a research funder (who will reinvest any profits in supporting future research and researchers). Only the latter options are both sustainable and contribute to the future of science, so the choice should be easy.

My most sincere thanks to Jordan, who brought Biology Open into existence and created a journal that gives back to the biological sciences community. I look forward to building on what Jordan started. Biology Open already provides an Open Access platform for publishing all aspects of biological research, from polar bears to cell polarity and everything in between. As a publication from the not-for-profit The Company of Biologists, it also already supports research and researchers around the world and reduces barriers to publication while maintaining scientific integrity. However, there is more to be done to improve the publication process and I very much look forward to the opportunity I now have as the new Editor-in-Chief of BiO.

## Supplementary Material

Supplementary information
