# Supplementary material for: The continuing evolution of publishing in the biological sciences
Source: Biol Open. 2018 Aug 15;7(8):bio037325. doi: 10.1242/bio.037325 (PMC6124566; doi:10.1242/bio.037325)
Supplement: Supplementary information [file biolopen-7-037325-s1.pdf]

Biology Open (2018): doi:10.1242/bio.037325: Supplementary information

Supplemental File 1 contains the results of the clustering analysis of abstract word content. It is a compressed archive that contains two files. Clusters_profiles.pdf contains the visual representation of the 8 clusters identified in the data. Cluster_words.xlsx is a Microsoft Excel spreadsheet that contains the words in each of these 8 clusters.

Click here to download Supplemental File 1

Biology Open • Supplementary information
